# Behavioral Intervention in Adolescents Improves Bone Mass, Yet Lactose Maldigestion Is a Barrier

**DOI:** 10.3390/nu10040421

**Published:** 2018-03-28

**Authors:** Yujin Lee, Dennis A. Savaiano, George P. McCabe, Francis M. Pottenger, Kathleen Welshimer, Connie M. Weaver, Linda D. McCabe, Rachel Novotny, Marsha Read, Scott Going, April Mason, Marta Van Loan, Carol J. Boushey

**Affiliations:** 1 Department of Nutrition Science, Purdue University, West Lafayette, IN 47907, USA; yujinlee1203@gmail.com (Y.L.); savaiano@purdue.edu (D.A.S.); weavercm@purdue.edu (C.M.W.); ldoyle@purdue.edu (L.D.M.); 2 Department of Statistics, Purdue University, West Lafayette, IN 47907, USA; mccabe@purdue.edu; 3 Curriculum Research and Development Group, University of Hawaii, Honolulu, HI 96822, USA; frankp@hawaii.edu; 4 Department of Health Education and Recreation, Southern Illinois University, Carbondale, IL 62901, USA; welshime@siu.edu; 5 Department of Human Nutrition, Food, and Animal Sciences, University of Hawaii, Honolulu, HI 96822, USA; novotny@hawaii.edu; 6 Graduate School, University of Nevada, Reno, NV 89557, USA; read@unr.edu; 7 Department of Nutritional Sciences, University of Arizona, Tucson, AZ 85721, USA; going@u.arizona.edu; 8 Kansas State University, Manhattan, KS 66506, USA; masona@k-state.edu; 9 Western Human Nutrition Research Center, USDA, Davis, CA 95616, USA; marta.vanloan@ars.usda.gov; 10 Epidemiology Program, University of Hawaii, Honolulu, HI 96813, USA

**Keywords:** adolescents, bone, calcium, perceived milk intolerance, lactose maldigestion

## Abstract

Calcium intake during adolescence is important for attainment of peak bone mass. Lactose maldigestion is an autosomal recessive trait, leading to lower calcium intake. The Adequate Calcium Today study aimed to determine if a school-based targeted behavioral intervention over one year could improve calcium intake and bone mass in early adolescent girls. The school-randomized intervention was conducted at middle schools in six states over one school year. A total of 473 girls aged 10–13 years were recruited for outcome assessments. Bone mineral content (BMC) was determined by dual energy X-ray absorptiometry. Dietary calcium intake was assessed with a semi-quantitative food frequency questionnaire. Baseline calcium intake and BMC were not significantly different between groups. After the intervention period, there were no differences in changes in calcium intake and BMC at any site between groups. An unanticipated outcome was a greater increase in spinal BMC among lactose digesters than lactose maldigesters in the intervention schools only (12 months) (6.9 ± 0.3 g vs. 6.0 ± 0.4 g, *p* = 0.03) and considering the entire study period (18 months) (9.9 ± 0.4 vs. 8.7 ± 0.5 g, *p* < 0.01). Overall, no significant differences between the intervention and control schools were observed. However, lactose digesters who received the intervention program increased bone mass to a greater extent than lactose maldigesters.

## 1. Introduction

The National Osteoporosis Risk Assessment identified Asian, Hispanic, and non-Hispanic white women as being at higher risk for osteoporosis compared with non-Hispanic Black women [1]. At least 90% of peak bone mass is achieved by age 18 years in girls [2]; thus, calcium intake during childhood and adolescence is an important factor in reaching peak bone mass in early adulthood.

According to the Institute of Medicine, the Dietary Reference Intake for calcium for adolescent girls is a Recommended Dietary Allowance of 1300 mg [3]. National surveys have demonstrated a decline in dietary calcium intake among girls as they progress through adolescence [4]. Dairy foods are known as an excellent source of calcium. Some barriers hindering the consumption of dairy foods are perceived milk intolerance (PMI), lactose intolerance, and lactose maldigestion (LM). Several studies have suggested an association between these barriers and low calcium intakes, and the incidence of osteoporosis [5,6,7,8,9,10]. 

Intervention studies using milk, calcium supplements, or calcium rich foods have been conducted to investigate a direct relationship between calcium intake and bone mass in prepubertal children [11,12,13,14]. Although the intervention amount of calcium has been varied, these studies have consistently demonstrated a positive association between calcium intake and bone mass in prepubertal children [11,12,13,14]. However, the benefits of the supplementation were rapidly lost after the intervention ceased [15]. The supplementation studies provided a short-term intervention that did not focus on changing food behaviors. In contrast, French et al. implemented a well-designed, group-randomized behavioral intervention to improve calcium intake among early adolescent girls [16]. The results revealed small, significant changes in calcium intake but no significant benefit to bone mass. Thus, the question remained of whether a behavioral intervention could have the same positive effect as supplementation. 

Our primary hypothesis was that early adolescent girls receiving a behavioral calcium intervention would increase calcium intake and improve bone mass compared with girls not receiving the intervention. The secondary hypothesis was that LM, as an obstacle to achieving adequate calcium intake, can be overcome by a behavioral intervention, resulting in changes in dietary calcium intake and bone mass among maldigesters that are similar to those of digesters. The study was designed to allow for examination of whether the intervention outcomes persisted past the intervention.

## 2. Materials and Methods 

### 2.1. Study Design and Sample Selection

**Figure 1:**
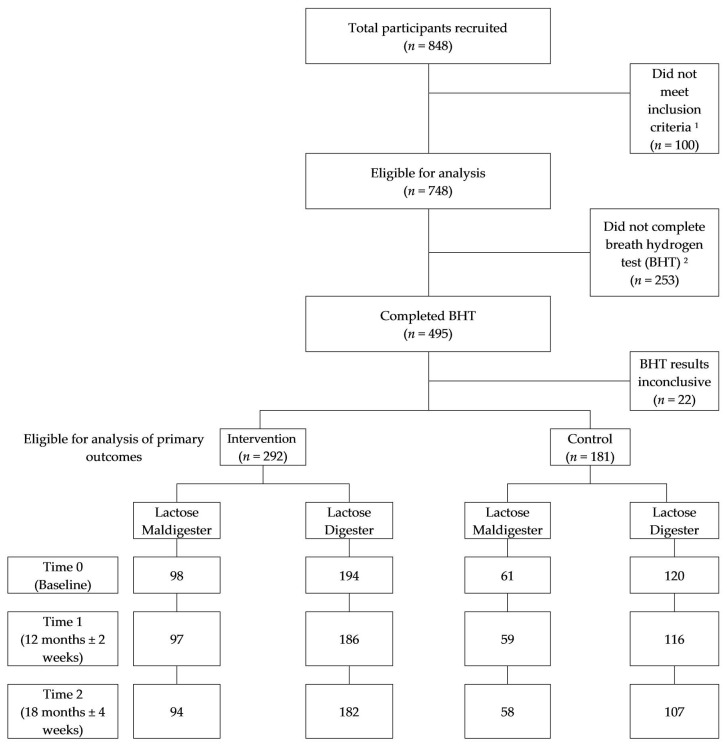
Consort diagram of participants included for analysis in the Adequate Calcium Today project; ^1^ Inclusion criteria of ethnic group not met; ^2^ Breath hydrogen test (BHT) was needed for classification of lactose maldigestion status

A school-randomized behavioral intervention study called the Adequate Calcium Today (ACT) project was conducted in sixth grade classrooms located in six states (Arizona, California, Hawaii, Indiana, Nevada, and Ohio) from September 2000 through September 2004. Middle schools within one-hour driving time of one of the designated dual energy X-ray absorptiometry (DXA) measurement sites were eligible to participate in the study if their student population had a higher proportion of Asian or Hispanic students than the respective state’s average. A recruitment goal for the total number schools was to enlist at least two pairs of schools within each state, for a total of 16 matched sets. Once schools agreed to participate, they were paired based on likeness in race/ethnic distribution and participation in free or reduced-price school lunches. Within a pairing, schools were randomly assigned to an intervention or a control group using the randomized complete block design. The randomization was done by the Data Coordinating Center at Purdue University. Outcomes assessors (i.e., DXA technicians) were blinded; however, participants could not be blinded to their group assignment. In some cases, two or more schools were recruited and pooled to reach adequate numbers and comparable ethnic distribution. The final groups consisted of seven pairs with two schools and seven pairs with three schools. No schools dropped out of the study during the project. The primary outcome measures were collected on a subset of girls in the sixth grade. Girls were recruited through posters, presentations and invitations mailed to all sixth-grade girls registered in the participating schools. Girls recruited for evaluation measures were limited to those identifying ancestry as Asian (at least 75%), Hispanic or non-Hispanic white, based on the race/ethnicity of their biological parents. Of the 848 girls recruited, 748 met the ethnic group criteria (Figure 1). The institutional review boards of the participating site in each state approved the study protocol, and written informed consent was obtained from the girls and their parents. At the beginning of the school term, prior to the first intervention lesson, girls were measured. The measurements were repeated at 12 months ± 2 weeks and 18 months ± 4 weeks after the first measure. This trial has been registered at www.clinicaltrials.gov (Identifier: NCT02202889).

### 2.2. Sample Size Estimation 

The sample size estimates were based on calculations from information in the literature available at the time that the study was designed [11,17]. The effects of a previous intervention were very similar for bone mineral content (BMC) and bone mineral density in a population very similar to the current study. Therefore, sample size calculations used 2.5% and 4.7% as the standard deviations in the percent change from baseline for total bone mineral density and lumbar spine density, respectively. With 75 individuals in each group (intervention and control, with three ethnic groups, this implied a total of 450 participants), we had at least an 80% chance of declaring the intervention successful when the difference in change of total bone mineral density from baseline was 1.16% or more; 90% when it was 1.33% or more. The comparable values for lumbar spine density were 2.17% and 2.51%.

### 2.3. Intervention Implementation

All sixth-grade boys and girls in the intervention schools received the intervention lessons; the boys received the intervention lessons in order to minimize disruption in the intervention schools. The control schools did not receive any intervention messages. Schools were asked to avoid participating in milk promotion campaigns during the intervention period. The ACT intervention program consisted of six sessions of 50 min each that were provided throughout the school year, with at least two-week intervals between sessions [18]. At school sites, the program was referred to as only “ACT” to minimize influence beyond the intervention. To insure consistency among sites, the sessions were conducted by trained research staff with classroom teachers present. The sessions were delivered via DVD (video portion) and CD (game portion). The objective of the program was to motivate changes in dietary behavior to increase the consumption of calcium rich foods, including traditional dairy sources and non-dairy sources relevant to Hispanic and Asian populations. The program used the ‘No Bones About It’ package which includes information about calcium and bone health, how to build skills for choosing calcium rich foods, motivational messages from role models, and opportunities for action planning and reflection. The intervention was developed based on a transtheoretical model [19]. According to research using this model [19], people pass through specific stages, each with its own characteristic cognitive, behavioral, and social processes and strategies, on their way to achieving and maintaining a behavior change. The intervention sessions were developed with two organizing logics: instructional logic [20] and behavioral stages logic [19]. Instructional logic is the classic approach to education in which basic concepts are introduced and proceed to more complex concepts. Bridging concepts are repeated for reinforcement. Behavioral stages logic is part of the transtheoretical model in which individuals undergo stages of readiness in accepting and adopting a new behavior.

### 2.4. Anthropometric Measures and Maturity

Anthropometric measurements included weight, standing and sitting heights, forearm and femur lengths, waist circumference, waist depth, and bitrochanteric width. All measurements were performed following the procedures in the Anthropometric Standardization Reference Manual [21], and details of the quality control methods used have been previously described [22]. For the purpose of this analysis, height and weight taken at baseline and changes in height and weight were used. Changes in height and weight were calculated as the height or weight at visit date (12 or 18 months) minus the height or weight at baseline. 

Breast and pubic hair pubertal staging was done using line drawings based on Tanner reference stages [23]. In a private setting, girls self-reported the stages on a scale of 1–5, 1 being prepubertal and 5 being fully mature for each area of breast and pubic hair. An average of breast and pubic hair scores was used for an average Tanner value. Postmenarcheal age was calculated as the visit date minus menarcheal date and divided by 365. For this analysis, postmenarcheal age was used as a measure of maturation.

### 2.5. LM Status and PMI Status 

LM was measured using a breath hydrogen test (BHT). Of the 748 eligible girls, 495 (66%) completed the BHT (Figure 1). The majority who did not complete BHT were from the Ohio site (*n* = 151). Details of the BHT used have been previously described by Matlik et al. [10]. As an initial standard, an increase of hydrogen >20 ppm above baseline was considered positive for LM. A constant elevation of hydrogen over the three-hour period was also considered positive for LM. The results for 22 girls who did not stabilize at a low concentration of hydrogen were considered indeterminate.

The PMI status and score were determined using methods described by Matlik et al. [10]. A questionnaire consisting of three statements was used: (1) I am allergic to milk; (2) I get a stomachache after drinking milk; and (3) I have been told that milk will make my stomach hurt after I drink it. Girls responded based on a scale of 1–5, 1 being “strongly disagree”, 5 being “strongly agree”; and “do not know” scored as missing. During several pilot tests with early adolescents, the statements were found to be satisfactory in terms of reliability by using test/retest (r = 0.53; *p* < 0.001; paired *t* test, *p* > 0.05) and internal consistency (Cronbach’s α = 0.68) analyses. An average of the responses was used for classifying PMI status if there were ≥2 non-missing responses. A score of >2 was defined to be indicative of PMI, as used previously [10]. 

### 2.6. Bone Mineral Assessment

At baseline and follow-up measurement visits, DXA [24] was used to determine BMC, bone mineral density, and bone area for total body, lumbar spine L2–L4, and left and right femurs. Measurements were performed following standardized procedures. Phantom measurements were reviewed independently at the University of California, San Francisco to determine within-laboratory and between-laboratory variance in DXA measurements. Details of bone measurements and quality control methods have been previously described [22]. In the present study, BMC for total body, lumbar spine L2–L4, total hip, and hip femoral neck were reported and analyzed. 

### 2.7. Dietary Assessment

At baseline and follow-up measurement visits, dietary calcium intakes from the past month were estimated using a semi-quantitative food frequency questionnaire (FFQ) developed for, and evaluated with, Asian, Hispanic, and non-Hispanic White youth [25,26]. Compared with two 24-h recalls, this questionnaire had high reliability (r = 0.68; *p* < 0.001) and accuracy (r = 0.54; *p* < 0.001). Daily calcium intakes from the 78 items listed in the FFQ were categorized in five ways. This included the intake of calcium exclusively from dairy foods (e.g., milk which is exclusively dairy calcium), and calcium intake from non-dairy foods (e.g., broccoli, tofu, soy milk, polenta, posole, miso). Another category was calcium intake from both dairy and non-dairy sources which was labeled as “calcium from mixed foods”. Examples of this grouping are pizza or cheese enchiladas where calcium from the cheese is from a dairy source and calcium from the crust/wrap, sauce, and any vegetables are from a non-dairy source. The category, “total dairy calcium”, was the sum of the calcium from the exclusive dairy foods and mixed foods groups. Finally, the category, “total dietary calcium”, was the sum of calcium from exclusive dairy foods, nondairy foods, and mixed foods groups. For the purposes of this analysis, total dietary calcium, total dairy calcium, and calcium exclusively from dairy foods were examined. Estimated total dietary calcium intakes that were <100 mg/day or >2500 mg/day were considered implausible and were excluded from analyses. 

### 2.8. Statistical Analyses

Data were analyzed using Statistical Package for the Social Sciences [27]. Before parametric analyses were done, probability plots were used to assess the normality of distribution. No variables required a transformation. Time periods examined were as follows: baseline to 12 months (influence of intervention), 12 months to 18 months (maintenance of intervention), and baseline to 18 months (overall influence of intervention). 

The primary outcome, change in BMC, was analyzed using a general linear regression. A common model was used for all bone sites (i.e., total body, spine L2–L4, total hip, and femoral neck). Treatment, LM status, PMI status, and race were considered to be fixed effects. Schools in the same state were paired based on comparable ethnic distribution and assigned as intervention or control schools. The school blocks were also nested within states, which made it impossible to separate out the two factors in the model. Therefore, state was eliminated from the model and school blocks were included in the model and considered to be a random effect. We excluded participants with missing values for LM or PMI (*n* = 275). Data for 473 girls were analyzed. Post-menarcheal age and state were used as covariates. Other covariates included were baseline value for each endpoint, baseline age, baseline height, change in height at 12 or 18 months, post-menarcheal age at 12 or 18 months, race, and state. 

A similar approach was used to examine the primary behavioral outcome variable: change in dietary calcium intake. Misreporters or outliers were eliminated prior to the analysis of changes in dietary calcium intake (*n* = 63). A common model was used for the three dietary calcium measures (i.e., total dietary calcium, total dairy calcium, and exclusive dairy calcium). Models in which a change in dietary calcium was examined included terms for treatment, LM status, PMI status, baseline value for each endpoint, baseline age, baseline weight, change in weight at 12 or 18 months, race, and school blocks. Baseline bone status and dietary calcium intake according to intervention status were examined by using two-sample *t* tests.

For the secondary outcome analyses, girls in the intervention group were further analyzed by LM status. Changes in BMC and dietary calcium intake were examined while adjusting for the aforementioned covariates. *p* < 0.05 and *p* < 0.10 were considered statistically significant and a trend towards significance, respectively.

## 3. Results

### 3.1. Descriptive Characteristics

The demographic, and physical and behavioral variables of participants by treatment group condition at baseline, 12 months, and 18 months are shown in Table 1. The retention rate was high throughout the study period. Of the 473 girls enrolled at baseline, 466 (98.5%) participated at 12 months and 438 (92.6%) participated at 18 months. At baseline, 181 girls and 292 girls were in the control and intervention groups, respectively. Race/ethnic groups were similarly represented: 31.7% Asian, 38.1% Hispanic, and 30.2% non-Hispanic White. However, more Asian girls and less Hispanic girls were in the intervention group than the control group. At baseline, the average age was 11.7 years and body mass index was 20.6 kg/m^2^. 

Of the 466 girls who completed a perceived milk intolerance questionnaire, only 64 girls (13.7%) perceived themselves as being milk intolerant, and there was no difference in the proportions across race/ethnic groups (X^2^ = 0.15, *df* = 2, *p* = 0.93). In the BHT, 159 of 473 girls (33.6%) were found to be lactose maldigesters. LM was more predominant in the Asian (*n* = 80) and Hispanic (*n* = 67) girls compared with Non-Hispanic White girls (*n* = 12) (X^2^ = 67.96, *df* = 2, *p* < 0.0001). Sixty-one lactose maldigesters were in the intervention group, and 98 lactose maldigesters were in the control group.

Of the 473 participants measured at baseline, 471 completed the FFQ, and data from 411 participants were included in the analysis after excluding outliers (*n* = 60). At follow-up visits at 12 and 18 months, completed FFQs were collected from 456 and 436 participants, respectively. Among the collected FFQs, data from 407 and 406 participants were included in the analyses after excluding outliers (*n* = 49 at 12 months and *n* = 30 at 18 months, respectively.) There were no statistical differences between the intervention and control groups with regard to physical development, age, and body mass index measured at baseline and follow-up visits.

### 3.2. Effects of the Intervention on Bone Mass Gains

Treatment-related changes in BMC for each bone site are shown in Table 2. There were no statistically significant group differences for changes in BMC for each site (*p* > 0.05). For the subgroup analyses, the participants in the intervention group were further analyzed to investigate the treatment effects based on LM status (Table 3). 

There was a greater increase in BMC in the spine (L2–L4) among lactose digesters (LD) compared with lactose maldigesters over a one-year intervention period (lactose maldigesters vs. lactose digesters: 6.0 ± 0.4 vs. 6.9 ± 0.3 g, *p* = 0.03) and during the entire 18-month study period (lactose maldigesters vs. lactose digesters: 8.7 ± 0.5 vs. 9.9 ± 0.4 g, *p* < 0.01). In addition, a similar trend was seen for the change in BMC at the hips (total) among lactose digesters compared with lactose maldigesters after 12 months of the intervention (lactose maldigesters vs. lactose digesters: 2.9 ± 0.2 vs. 3.3 ± 0.2 g, *p* = 0.09). Changes in BMC at other sites between LM and LD were not significantly different. In addition, for the participants in the control group, there were no statistically significant differences in changes in BMC at all sites based on lactose digestion status (See Appendix A, Table A1 and Table A2).

For the subgroup analyses based on PMI status, there were no differences between girls with PMI and without PMI with regard to changes in BMC at all sites (data not shown).

### 3.3. Effects of the Intervention on Dietary Calcium Intake

Changes in dietary calcium intake based on treatment group are shown in Table 2. There were no significant differences in dietary calcium intake between girls in the intervention group and those in the control group. Table 3 shows changes in dietary calcium intake in the intervention group based on lactose digestion status. There were no differences in changes in dietary calcium intake between lactose maldigesters and lactose digesters.

## 4. Discussion

The results of the present study show that a school-randomized behavioral intervention did not have any beneficial effect on dietary calcium intake and bone accretion among early adolescent girls. On the other hand, there was a greater increase in spinal (L2–L4) bone mass over a 12-month period and an 18-month period among lactose digesters in the intervention group compared with lactose maldigesters in the intervention group. Thus, some benefit appears to have accrued in lactose digesters in the behavioral intervention group; however, among the lactose maldigesters no benefit to bone mass was seen. 

These results were not consistent with the Cal-Girls study [16], which included a physical activity component. No significant difference in change in bone mass was found in the Cal-Girls study, whereas dietary calcium intake significantly increased over the two-year intervention period. In the current study, the semi-quantitative food frequency questionnaire was specifically designed and evaluated for use with Asian, Hispanic, and non-Hispanic white adolescents [25,26]. A study by Horner et al. [28] among adult women concluded participants that underreport energy intake by 20.8% when using a comprehensive food frequency questionnaire. Thus, despite the intensive development of the calcium food frequency questionnaire used in the present study, the calcium intakes of participants may have included enough error to result in no differences in dietary calcium intake between the intervention and control groups. The Cal-girls study [16] used a single 24-h dietary recall, while DeBar and colleagues [29] used multiple days of dietary records and detected a significant change in calcium intake. Different dietary assessment tools among the studies may account for the discrepancies between studies. Furthermore, Golden et al. [30] suggested weight-bearing activity and vitamin D status beneficially influence bone mineral accrual in children and adolescents. It is possible that weight-bearing activity and vitamin D status may play a role in bone accretion; however, data for these factors were not collected in the present study. 

More Asians were in the intervention group compared with the control group. Asians may have lower calcium intakes and bone mass compared with other ethnic groups [31]. Thus, it might be expected that having more Asians in the intervention group would influence the difference between dietary calcium intake and bone mass for the intervention and control groups. The FFQ used to assess calcium intake contained unique calcium food sources traditional to Asian diets and often missing in most dietary assessment tools used in the US [25,26]. However, a cross-sectional study of differences and predictors of bone mass among this sample of girls found that Asian girls have a smaller skeleton than white or Hispanic girls. Any racial/ethnic differences in BMC were found to be mostly due to smaller size [22]. Additional work investigating variability in bone geometry among this same sample of Asian, Hispanic, and non-Hispanic white girls concluded that ethnicity was not a significant predictor of bone cross sectional area and section modulus [32]. After controlling for body size and pubertal maturation, there were no differences between the ethnic groups in bone geometry. These observations may help explain why, despite the partial ethnic group imbalance, there were no differences in dietary calcium intake or bone mass between the intervention and control groups at baseline, as we adjusted for known confounders.

The intervention program had a greater positive impact on bone mass for lactose digesters compared with lactose maldigesters. Lactose maldigestion is an autosomal recessive trait which often leads to lower calcium intake compared with lactose digesters [5,6,7,9], which may have resulted in the greater increase in lumbar spine BMC in lactose digesters compared to lactose maldigesters. In the current study, the baseline results from two sites (California and Indiana) were previously reported by Matlik et al. [10]. Among this sample, the prevalence of PMI was highest among the non-Hispanic white girls (21.4%) compared to the Asian or Hispanic girls, at 19.6% and 15.8%, respectively. The results showed that girls with PMI consumed less food calcium and had lower spinal BMC than those without PMI. However, after inclusion of all sites and receiving a one-year intervention program, there were no differences in changes in bone mass and dietary calcium intake between girls with PMI and without PMI. This finding may suggest that girls with PMI may have overcome this psychological barrier through the behavioral intervention. A decline in calcium intake is characteristic of this age group [4]. In the intervention group, there was an attenuated reduction among the lactose digesters of exclusively dairy calcium compared to the lactose maldigesters. The difference was not statistically significant, however may be noteworthy.

The strengths of the current study include its large sample size representing locations throughout the United States, high level of retention and the uniqueness of examining the influence of lactose digestion and PMI status on dietary calcium intake and bone mass under the intervention. There were some limitations in this study. The sample may have been biased because drinking a glass of milk was a requirement of recruitment; thus, it is possible that participants were more likely to be milk drinkers, which contributed to the high baseline level of calcium intake. In addition, physical activity is known as a contributor to bone development; however, the results from the physical activity questionnaire used in this study played no significant role in modifying the outcomes [33,34]. Therefore, this variable was not used in any of the models. The extensive FFQ was developed exclusively for the assessment of calcium intake; thus, the influence of other nutrients important for bone formation, such as vitamin D, magnesium, and vitamin K were not evaluated. Our results are also limited in that we did not control for long term UVA and UVB exposure, and serum 25(OH)D status was not included.

## 5. Conclusions

A school-based behavioral intervention program did not have any positive effect on dietary calcium intake and bone mass in early adolescent girls aged 10 to 13 years. An unexpected finding among the intervention group only was that lactose digesters had a greater increase in BMC in the lumbar spine (L2–L4) than lactose maldigesters. Therefore, future calcium intervention trials among early adolescents should consider LM status as a potential confounder of bone mass accretion in this age group. 

## Figures and Tables

**Table 1:** Demographic and physiological variables among early adolescent girls by treatment group at baseline and follow-up.

	Baseline		12 Months		18 Months	
Group ^a^	C	I	C	I	C	I
Total Participants (*n*)	181	292	176	290	164	274
Race/Ethnic Group (*n*)						
Asian (%)	45 (25)	105 (36)	43 (24)	104 (36)	43 (26)	104 (38)
Hispanic (%)	81 (45)	99 (34)	78 (44)	98 (34)	68 (41)	86 (31)
Non-Hispanic White (%)	55 (30)	88 (30)	55 (32)	88 (30)	53 (33)	84 (31)
Location (*n*)						
Arizona	51	71	49	70	39	56
California	39	81	38	80	38	78
Hawaii	30	81	29	81	29	81
Indiana	50	50	49	50	47	50
Nevada	11	9	11	9	11	9
Physical Development ^b^						
Tanner Score	2.43 ± 0.97	2.41 ± 0.82	3.13 ± 0.97	3.09 ± 0.97	3.32 ± 0.91	3.30 ± 0.88
Tanner Upper	2.25 ± 1.01	2.25 ± 0.87	2.89 ± 1.07	2.88 ± 1.09	3.07 ± 1.03	3.06 ± 0.98
Tanner Lower	2.61 ± 1.12	2.58 ± 1.01	3.37 ± 1.08	3.33 ± 1.04	3.58 ± 0.94	3.55 ± 1.05
Age ^b^ (years)	11.7 ± 0.44	11.7 ± 0.46	12.8 ± 0.44	12.7 ± 0.46	13.3 ± 0.45	13.2 ± 0.47
BMI ^b^ (kg/m^2^)	20.6 ± 4.56	20.5 ± 4.70	21.5 ± 4.79	21.4 ± 5.08	21.9 ± 4.77	21.9 ± 5.18
Complete PMI ^c^ questionnaire	179	287	-	-	-	-
Positive for PMI ^c^	22	42	21	42	20	39
Completed BHT ^c^	181	292	-	-	-	-
Lactose maldigester ^d^ (%)	61 (34)	98 (34)	59 (34)	98 (34)	58 (35)	93 (34)
Completed FFQ ^c^	181	290	174	282	163	273
FFQ excluding outliers ^e^	160	251	157	250	153	253

^a^ Differences between intervention and control groups (I = intervention; C = Control); values are means ± SDs and were obtained using the two-sample *t* test in SPSS; no statistical difference between control and intervention groups (*p* > 0.05); ^b^ data are means ± standard deviations; ^c^ perceived milk intolerance (PMI), breath hydrogen test (BHT), food frequency questionnaire (FFQ); ^d^ based on completed breath hydrogen test measured at baseline; ^e^ based on completed food frequency questionnaires. Girls with dietary calcium intakes <100 mg or >2500 mg were excluded as outliers.

**Table 2:** Calcium intake and bone mass among early adolescent girls by treatment group at baseline and change at follow-up visits ^a.^

	Control				Intervention				Group Differences ^b^, *p*		
	Baseline	Δ0–12 Months	Δ12–18 Months	Δ0–18 Months	Baseline	Δ0–12 Months	Δ12–18 Months	Δ0–18 Months	Δ0–12 Months	Δ12–18 Months	Δ0–18 Months
Dietary calcium intake ^c^, mg											
Total dietary calcium	1123 ± 56	−92 ± 64	−40 ± 64	−76 ± 70	1052 ± 566	−39 ± 51	−89 ± 54	−188 ± 54	0.42	0.47	0.17
Total dairy calcium	947 ± 489	−85 ± 57	−32 ± 59	−72 ± 62	895 ± 521	−39 ± 45	−75 ± 49	−176 ± 48	0.45	0.49	0.16
Exclusively dairy calcium	650 ± 390	−87 ± 48	−20 ± 51	−59 ± 55	632 ± 416	−51 ± 38	−22 ± 43	−131 ± 42	0.51	0.97	0.28
Bone mineral content ^d^, g											
Total body	1595 ± 372	293 ± 19	108 ± 14	388 ± 21	1585 ± 397	275 ± 15	107 ± 12	390 ± 17	0.45	0.92	0.93
Total hip	24 ± 5	2.8 ± 0.2	0.9 ± 0.1	3.8 ± 0.2	23 ± 5	3.1 ± 0.2	0.9 ± 0.1	4.2 ± 0.2	0.20	0.51	0.08
Spine (L2–L4)	28 ± 7	6.4 ± 0.4	2.6 ± 0.2	9.0 ± 0.4	28 ± 8	6.4 ± 0.3	2.6 ± 0.2	9.3 ± 0.3	0.92	0.95	0.58
Femoral neck	4 ± 0.7	0.47 ± 0.03	0.20 ± 0.02	0.66 ± 0.04	4 ± 0.7	0.46 ± 0.02	0.17 ± 0.02	0.66 ± 0.03	0.91	0.29	0.99

^a^ Values are means ± standard deviations for group differences at baseline from two-sample *t* tests; values are means ± standard errors for changes in calcium intake and bone mass based on a general linear model; ^b^ group differences represent the differences between intervention and control groups; ^c^ model was adjusted for lactose maldigestion (LM), perceived milk intolerance (PMI), school blocks, race, baseline value for outcome, baseline age, baseline weight, and change in weight at 12 or 18 months; girls with dietary calcium intakes <100 mg or >2500 mg were excluded as outliers and were removed from analysis; ^d^ model was adjusted for LM, PMI, school blocks, race, baseline value for outcome, baseline age, baseline height, change in height at 12 or 18 months, and post-menarcheal age.

**Table 3:** Calcium intake and bone mass among early adolescent girls in the intervention group based on lactose digestion status at baseline and change at follow-up visits ^a.^

	Lactose Maldigesters				Lactose Digesters				Group Differences ^b^, *p*		
	Baseline	Δ0–12 Months	Δ12–18 Months	Δ0–18 Months	Baseline	Δ0–12 Months	Δ12–18 Months	Δ0–18 Months	Δ0–12 Months	Δ12–18 Months	Δ0–18 Months
Dietary calcium intake ^c^, mg											
Total dietary calcium	1126 ± 580	−53 ± 74	−165 ± 77	−209 ± 77	1014 ± 557	12 ± 59	−75 ± 61	−151 ± 61	0.39	0.27	0.46
Total dairy calcium	957 ± 530	−55 ± 66	−149 ± 71	−204 ± 68	864 ± 516	−5 ± 52	−57 ± 56	−148 ± 54	0.46	0.29	0.42
Exclusively dairy calcium	653 ± 399	−59 ± 55	−84 ± 64	−156 ± 61	622 ± 425	−41 ± 43	12 ± 51	−110 ± 48	0.74	0.16	0.45
Bone mineral content ^d^, g											
Total body	1535 ± 392	273 ± 22	108 ± 15	393 ± 24	1610 ± 398	279 ± 17	115 ± 12	404 ± 20	0.76	0.63	0.65
Total hip	22 ± 5	2.9 ± 0.2	0.9 ± 0.1	4.2 ± 0.2	23 ± 5	3.3 ± 0.2	1.0 ± 0.1	4.4 ± 0.2	0.09	0.51	0.33
Spine (L2–L4)	27 ± 8	6.0 ± 0.4	2.3 ± 0.2	8.7 ± 0.5	28 ± 8	6.9 ± 0.3	3.0 ± 0.2	9.9 ± 0.4	0.03 *	<0.01 *	<0.01 *
Femoral neck	3 ± 1	0.43 ± 0.04	0.16 ± 0.02	0.64 ± 0.04	4 ± 0.7	0.49 ± 0.03	0.19 ± 0.02	0.70 ± 0.03	0.09	0.16	0.14

^a^ Values are means ± standard deviations for group differences at baseline from two-sample *t* tests; values are means ± standard errors for changes in calcium intake and bone mass based on a general linear model; ^b^ group differences represent the differences between lactose digesters and lactose maldigesters; ^c^ model was adjusted for lactose maldigestion (LM), perceived milk intolerance (PMI), school blocks, race/ethnicity, baseline value for outcome, baseline age, baseline weight, and change in weight at 12 or 18 months; girls with dietary calcium intakes <100 mg or >2500 mg were excluded as outliers and were removed from analysis; ^d^ model was adjusted for LM, PMI, school blocks, race/ethnicity, baseline value for outcome, baseline age, baseline height, change in height at 12 or 18 months, and post-menarcheal age; * *p* < 0.05.

## Appendix A

**Table A1:** Calcium intake and bone mass among early adolescent girls in the control group based on lactose digestion status at baseline and change at first follow-up visit at 12 months.

	**Baseline ^a^**		**12 Months ^a^**		**Adjusted Differences between Girls in Maldigester and Digester Groups**	**95% Confidence Interval**	**Group Difference, *p***
	**Lactose Maldigester (*n* = 55)**	**Lactose Digester (*n* = 105)**	**Lactose Maldigester (*n* = 53)**	**Lactose Digester (*n* = 105)**			**Δ0–12 Months**
Dietary calcium intake, mg ^b^							
Total dietary calcium	1162 ± 566	1102 ± 556	958 ± 475	1023 ± 518	95	−94 to 285	0.321
Total dairy calcium	956 ± 494	942 ± 488	788 ± 417	870 ± 456	80	−88 to 248	0.349
Exclusively dairy calcium	646 ± 415	652 ± 379	506 ± 327	607 ± 367	37	−102 to 177	0.597
	**Baseline ^a^**		**12 Months ^a^**		**Adjusted Differences between Girls in Maldigester and Digester Groups**	**95% Confidence Interval**	**Group Difference, *p***
	**Maldigester (*n* = 61)**	**Digester (*n* = 120)**	**Maldigester (*n* = 59)**	**Digester (*n* = 117)**			**Δ0–12 Months**
Bone mineral content, g ^c^							
Total body	1546 ± 368	1619 ± 374	1804 ± 416	1903 ± 390	9.29	−40.24 to 58.81	0.710
Total hip	23.11 ± 4.77	23.72 ± 4.99	25.60 ± 4.84	26.83 ± 5.02	−0.41	−0.96 to 0.13	0.136
Spine (L2–L4)	27.39 ± 7.13	27.83 ± 7.12	33.06 ± 7.67	34.15 ± 7.98	−0.42	−1.45 to 0.60	0.411
Femoral neck	3.52 ± 0.66	3.62 ± 0.68	3.89 ± 0.67	4.07 ± 0.74	−0.08	−0.17 to 0.01	0.09

^a^ Values are means ± standard deviations; ^b^ model was adjusted for lactose maldigestion (LM), perceived milk intolerance (PMI), school blocks, race/ethnicity, baseline value for outcome, baseline age, baseline weight, and change in weight at 12 or 18 months; girls with dietary calcium intakes <100 mg or >2500 mg were excluded as outliers and were removed from analysis; ^c^ model was adjusted for LM, PMI, school blocks, race/ethnicity, baseline value for outcome, baseline age, baseline height, change in height at 12 or 18 months, and post-menarcheal age.

**Table A2:** Calcium intake and bone mass among early adolescent girls in the control group based on lactose digestion status at 12 months and change at second follow-up visit at 18 months.

	**12 Months ^a^**		**18 Months ^a^**		**Adjusted Differences between Girls in Maldigester and Digester Groups**	**95% Confidence Interval**	**Group Difference, *p***
	**Lactose Maldisgeter (*n* = 53)**	**Lactose Digester (*n* = 105)**	**Lactose Maldigester (*n* = 53)**	**Lactose Digester (*n* = 102)**			**Δ12–18 Months**
Dietary calcium intake, mg ^b^							
Total dietary calcium	958 ± 475	1023 ± 518	1001± 611	976 ± 469	107	−124 to 339	0.361
Total dairy calcium	788 ± 417	870 ± 456	822 ± 562	840 ± 418	82	−125 to 290	0.433
Exclusively dairy calcium	506 ± 327	607 ± 367	540 ± 430	596 ± 368	72	−95 to 238	0.395
	**12 months ^a^**		**18 months ^a^**		**Adjusted Differences between Girls in Maldigester and Digester Groups**	**95% Confidence Interval**	**Group Difference, *p***
	**Lactose Maldisgeter (*n* = 59)**	**Lactose Digester (*n* = 117)**	**Lactose Maldisgeter (*n* = 57)**	**Lactose Digester (*n* = 106)**			**Δ12–18 Months**
Bone mineral content, g ^c^							
Total body	1804 ± 416	1903 ± 390	1899 ± 388	2027 ± 391	−43.10	−88.06 to 1.87	0.060
Total hip	25.60 ± 4.84	26.83 ± 5.02	26.57 ± 4.76	28.10 ± 4.99	−0.04	−0.44 to 0.36	0.837
Spine (L2–L4)	33.06 ± 7.67	34.15 ± 7.98	35.59 ± 7.23	36.83 ± 8.10	0.11	−0.61 to 0.83	0.762
Femoral neck	3.89 ± 0.67	4.07 ± 0.74	4.05 ± 0.68	4.28 ± 0.75	−0.03	−0.10 to 0.04	0.354

^a^ Values are means ± standard deviations; ^b^ model was adjusted for lactose maldigestion (LM), perceived milk intolerance (PMI), school blocks, race/ethnicity, baseline value for outcome, baseline age, baseline weight, and change in weight at 12 or 18 months; girls with dietary calcium intakes <100 mg or >2500 mg were excluded as outliers and were removed from analysis; ^c^ model was adjusted for LM, PMI, school blocks, race/ethnicity, baseline value for outcome, baseline age, baseline height, change in height at 12 or 18 months, and post-menarcheal age.
